# Phenotypic subtypes of fibrotic hypersensitivity pneumonitis identified by machine learning consensus clustering analysis

**DOI:** 10.1186/s12931-024-02664-x

**Published:** 2024-01-18

**Authors:** Tananchai Petnak, Wisit Cheungpasitporn, Charat Thongprayoon, Tulaton Sodsri, Supawit Tangpanithandee, Teng Moua

**Affiliations:** 1 Division of Pulmonary and Pulmonary Critical Care Medicine, Faculty of Medicine Ramathibodi Hospital, Mahidol University, Nakhon Pathom, Thailand; 2 Division of Nephrology and Hypertension, Mayo Clinic, Rochester, MN United States; 3 Faculty of Medicine Ramathibodi Hospital, Chakri Naruebodindra Medical Institute, Mahidol University, Samut Prakan, Thailand; 4 Division of Pulmonary and Critical Care Medicine, Mayo Clinic, 200 First St SW, Rochester, MN 55905 United States

**Keywords:** Fibrotic hypersensitivity pneumonitis, Machine learning cluster analysis, Survival

## Abstract

**Background:**

Patients with fibrotic hypersensitivity pneumonitis (f-HP) have varied clinical and radiologic presentations whose associated phenotypic outcomes have not been previously described. We conducted a study to evaluate mortality and lung transplant (LT) outcomes among clinical clusters of f-HP as characterized by an unsupervised machine learning approach.

**Methods:**

Consensus cluster analysis was performed on a retrospective cohort of f-HP patients diagnosed according to recent international guideline. Demographics, antigen exposure, radiologic, histopathologic, and pulmonary function findings along with comorbidities were included in the cluster analysis. Cox proportional-hazards regression was used to assess mortality or LT risk as a combined outcome for each cluster.

**Results:**

Three distinct clusters were identified among 336 f-HP patients. Cluster 1 (*n* = 158, 47%) was characterized by mild restriction on pulmonary function testing (PFT). Cluster 2 (*n* = 46, 14%) was characterized by younger age, lower BMI, and a higher proportion of identifiable causative antigens with baseline obstructive physiology. Cluster 3 (*n* = 132, 39%) was characterized by moderate to severe restriction. When compared to cluster 1, mortality or LT risk was lower in cluster 2 (hazard ratio (HR) of 0.42; 95% CI, 0.21–0.82; *P* = 0.01) and higher in cluster 3 (HR of 1.76; 95% CI, 1.24–2.48; *P* = 0.001).

**Conclusions:**

Three distinct phenotypes of f-HP with unique mortality or transplant outcomes were found using unsupervised cluster analysis, highlighting improved mortality in fibrotic patients with obstructive physiology and identifiable antigens.

**Supplementary Information:**

The online version contains supplementary material available at 10.1186/s12931-024-02664-x.

## Background

Hypersensitivity pneumonitis (HP) is an immune-mediated interstitial lung disease characterized by injury from inhaled organic or inorganic antigens [1, 2]. The 2020 ATS/JRS/ALAT clinical practice guideline categorizes HP into fibrotic and non-fibrotic subtypes based on radiologic or histopathologic findings [1]. Patients with fibrotic hypersensitivity pneumonitis (f-HP) have worse survival compared to non-fibrotic with an all-cause mortality rate of 67.5 per 1000 person-years [3]. Identification and avoidance of causative antigens has recently been described as associated with better survival in those with fibrotic disease [4]. Exposure type (e.g., avian vs. mold vs. bacterial) may also be associated with differential outcomes [4]. Specific radiologic findings among patients with lung fibrosis may be correlated with lower forced vital capacity (FVC) or lung function [5]. Although multiple studies have reported the association of specific clinical domains with survival in f-HP, concomitant domains or phenotype analyses have not been previously described.

Machine learning and artificial intelligence have advanced the diagnostic and prognostic association of clinical parameters in medicine. Prior cohort studies have found specific variables are associated with outcome, though have not incorporated them into phenotypic subgroups or structuring. An additional benefit of phenotyping may be tailoring treatments according to subgroup characteristics, particularly in the context of heterogeneously presenting diseases like HP. Recent studies have shown that clustering methodology may differentiate unique phenotypes with distinct clinical courses or outcomes [6–8]. We conducted a study using unsupervised machine learning to identify clinical phenotypes in f-HP and assess their comparative mortality and transplant risk.

## Methods

### Subject selection

This study is a single-center retrospective cohort conducted at Mayo clinic Rochester. Suspected f-HP patients diagnosed between January 2005 and December 2020 were identified using a computer-assisted search. Each medical record was reviewed by study investigators to verify exposure history, serum specific IgG testing, radiologic findings, bronchoalveolar lavage analysis, and histopathology if obtained. Patients were identified as having identifiable causative antigens if there was documentation of suspected environmental exposure regardless of serum specific IgG testing. Final diagnosis of f-HP was based on the 2020 ATS/JRS/ALAT clinical practice guideline [1] highlighting specific levels of diagnostic confidence. Diagnoses were categorized as definite (level of confidence ≥ 90%), high (80–89%), moderate (70–79%), or low confidence (51–69%). Patients with diagnostic confidence < 50% or missing baseline pulmonary function testing (PFT) were excluded. Our study was approved by Mayo Clinic Institutional Review Board (approval No. 20–000211).

### Data collection

In addition to diagnostic variables, age, sex, smoking status, body mass index (BMI), presenting PFTs as percent predicted findings for total lung capacity (TLC%), forced vital capacity (FVC%), forced expiratory volume in the first second (FEV1%), FEV1/FVC ratio, diffusion capacity for carbon monoxide (DL_CO_%), and selected comorbidities (see Table 1) were collated. Missing non-PFT data were imputed by the Random Forest method [9]. Radiologic findings included presence of mosaic attenuation, honeycombing, and those with probable or consistent usual interstitial pneumonia (UIP) high resolution computed tomography (HRCT) patterns. Dates of death, LT, or last follow-up were used to assess long-term outcomes.

**Table 1:** Baseline characteristics of fibrotic hypersensitivity pneumonitis patients as classified by cluster

Variables	All (*N* = 336)	Cluster 1 (*N* = 158)	Cluster 2 (*N* = 46)	Cluster 3 (*N* = 132)	*P* value
Age, years	65.3 ± 10.9	68.0 ± 9.7	60.9 ± 12.5	63.5 ± 10.9	< 0.001
Male	160 (47.6)	73 (46.2)	21 (45.7)	66 (50.0)	0.78
Ever smoking	147 (43.8)	72 (45.6)	17 (37.0)	58 (43.9)	0.59
BMI, kg/m^2^	31.3 ± 6.6	31.6 ± 6.1	27.5 ± 5.8	32.1 ± 7.0	< 0.001
HRCT pattern					0.14
- Typical HP - Compatible with HP - Indeterminate for HP	222 (66.0) 59 (17.6) 55 (16.4)	103 (65.2) 26 (16.5) 29 (18.4)	36 (78.3) 8 (17.4) 2 (4.4)	83 (62.9) 25 (18.9) 24 (18.2)	
Histopathologic findings					0.06
- No tissue biopsy - HP - Probable HP - Indeterminate for HP	107 (31.9) 134 (39.9) 39 (11.6) 56 (16.6)	60 (38.0) 59 (37.4) 16 (10.1) 23 (14.5)	18 (39.1) 19 (41.3) 3 (6.5) 6 (13.1)	29 (22.0) 56 (42.4) 20 (15.2) 27 (20.4)	
Diagnostic confidence					0.04
- Definite diagnosis - High confidence - Moderate confidence - Low confidence	133 (39.6) 31 (9.2) 101 (30.1) 71 (21.1)	57 (36.1) 13 (8.2) 51 (32.3) 37 (23.4)	22 (47.8) 0 (0.0) 17 (37.0) 7 (15.2)	54 (40.9) 18 (13.6) 33 (25.0) 27 (20.5)	
Identifiable causative antigen					
- Any exposure - Bird proteins - Farm environment - Domestic antigens - Hot tub/sauna - Other specific environments - Multiple exposures	202 (60.1) 117 (34.8) 47 (14.0) 50 (14.9) 8 (2.4) 9 (2.7) 47 (14.0)	99 (62.7) 60 (38.0) 25 (15.8) 23 (15.6) 2 (1.3) 3 (1.9) 21 (13.3)	39 (84.8) 23 (50.0) 7 (15.2) 7 (15.2) 5 (10.9) 1 (2.2) 8 (17.4)	64 (48.5) 34 (25.8) 15 (11.4) 20 (15.2) 1 (0.8) 5 (3.8) 18 (13.6)	< 0.001 0.006 0.53 1.00 0.003 0.66 0.76
Positive serum specific IgG					
- Any specific IgG - IgG against bird proteins - IgG against mold - IgG against bacteria	148 (44.1) 101(30.1) 73 (21.7) 27 (8.0)	63 (39.9) 45 (28.5) 31 (19.6) 8 (5.1)	32 (69.6) 24 (52.2) 13 (28.3) 6 (13.0)	53 (40.2) 32 (24.2) 29 (22.0) 13 (9.9)	0.001 0.002 0.43 0.11
Identifiable *nontuberculous mycobacterium*	4 (1.2)	1 (0.6)	2(4.4)	1 (0.8)	0.15
Identifiable causative exposures confirmed by serum specific IgG	93 (27.7)	41 (26.0)	24 (52.2)	28 (21.2)	< 0.001
Mosaic attenuation on HRCT	280 (83.3)	129 (81.7)	43 (93.5)	108 (81.8)	0.13
Honeycombing cysts on HRCT	61 (18.2)	29 (18.4)	5 (10.9)	27 (20.5)	0.36
UIP pattern on HRCT	21 (6.3)	10 (6.3)	0 (0)	11 (8.33)	0.11
Baseline pulmonary function test, %predicted					
- FVC - FEV_1_ - FEV_1_/FVC ratio* - FEF_25 − 75%_ - DL_CO_ - TLC - RV - RV/TLC ratio	65.6 ± 16.7 68.6 ± 17.5 0.81 ± 0.09 90.4 ± 43.6 50.0 ± 15.5 72.5 ± 15.8 82.9 ± 32.4 111.7 ± 27.0	78.2 ± 10.9 83.2 ± 10.5 0.82 ± 0.06 114.5 ± 43.2 55.8 ± 13.9 78.4 ± 10.7 78.2 ± 17.9 97.5 ± 14.8	63.8 ± 16.1 57.8 ± 14.6 0.69 ± 0.13 44.5 ± 25.1 57.2 ± 16.6 90.8 ± 16.3 139.9 ± 42.3 151.8 ± 30.5	51.1 ± 9.0 54.8 ± 9.2 0.83 ± 0.06 77.4 ± 28.3 40.5 ± 11.6 59.2 ± 8.6 68.6 ± 17.5 114.8 ± 21.6	< 0.001 < 0.001 < 0.001 < 0.001 < 0.001 < 0.001 < 0.001 < 0.001
Co-morbidities					
- Diabetes mellitus - Hypertension - COPD - Gastroesophageal reflux - Coronary arterial disease - OSA - Pulmonary hypertension - Anxiety/depression	44 (13.1) 114 (33.9) 46 (13.7) 76 (22.6) 71 (21.1) 94 (28.0) 63 (18.8) 32 (9.5)	21 (13.3) 57 (36.1) 24 (15.2) 44 (27.9) 41 (26.0) 56 (35.4) 17 (17.1) 18 (11.4)	4 (8.7) 12 (26.1) 7 (15.2) 7 (15.2) 7 (15.2) 5 (10.9) 5 (10.9) 2 (4.4)	19 (14.4) 45 (34.1) 15 (11.4) 25 (18.9) 23 (17.2) 33 (25.0) 31 (13.5) 12 (9.1)	0.68 0.46 0.59 0.09 0.13 0.002 0.14 0.40
Treatment (throughout the follow-up period)					
- No treatment - Corticosteroids - Steroid-sparing agent - Antigen avoidance	42 (12.5) 288 (85.7) 133 (39.6) 114 (33.9)	21 (13.3) 134 (84.8) 59 (37.3) 59 (37.3)	12 (26.1) 34 (73.9) 11 (23.9) 23 (50)	9 (6.8) 120 (90.9) 63 (47.7) 32 (24.2)	0.003 0.02 0.01 0.004

* Data are presented as absolute ratio of FEV_1_ to FVC

Data are presented as mean ± SD and number (percentage) for continuous and categorical data, respectively

BMI, body mass index; COPD, chronic pulmonary obstructive disease; DL_CO_, diffuse capacity for carbon monoxide; FEF_25 − 75%,_ forced expiratory flow at 25–75% of forced vital capacity; FEV_1_, forced expiratory volume in 1 s; FVC, forced vital capacity; HP, hypersensitivity pneumonitis; HRCT, high-resolution computed tomography; OSA, obstructive sleep apnea; RV, residual volume; TLC, total lung capacity; UIP, usual interstitial pneumonia

### Clustering analysis

We used an unsupervised machine learning consensus clustering approach to identify clinical subtypes of patients with f-HP [10]. A pre-specified subsampling parameter of 80% with 100 iterations was pursued. The number of potential clusters (k) was set to a range of two to ten to avoid excessive cluster numbers and clinically irrelevant groupings. The optimal number of clusters was determined by a consensus matrix (CM) heat map, cumulative distribution function (CDF), cluster-consensus plots in the within-cluster consensus scores, and proportion of ambiguously clustered (PAC) pairs. The within-cluster consensus score, an average consensus value for all pairs of individuals in the same cluster, ranged between 0 and 1 [11]. A value closer to 1 indicated better cluster stability. PAC, ranging between 0 and 1, was defined as the proportion of all sample pairs with consensus values falling within the predetermined boundaries [12]. A value closer to zero indicated better cluster stability [12]. Additional details of consensus clustering algorithms are described in the Supplementary file.

### Statistical analysis

After cluster identification, we compared baseline characteristics between each cluster using analysis of variance (ANOVA) and Chi-square for continuous and categorical variables, respectively. The standardized mean differences of clinical characteristics between each cluster and the whole cohort was used to determine specific clinical characteristics for each cluster. Variables with an absolute standardized mean difference of > 0.3 were considered key characteristics of the cluster.

Association of each cluster with transplant-free survival was evaluated using Cox proportional hazard regression analysis reported as a hazard ratio (HR) with 95% confidence interval (CI). Survival status and lung transplantation were ascertained through medical record review and cross-matched with a United States Social Security Death Index (USSDI) search. Since all baseline characteristics were considered for cluster development, we did not adjust for specific variables in the model. *P* values of < 0.05 were considered statistically significant. All analyses were performed using R, version 4.0.3 (RStudio, Inc., Boston, MA, USA), with the ConsensusClusterPlus package (version 1.46.0) for consensus clustering analysis and the missForest package for imputation of missing data [9].

## Results

**Fig. 1:**
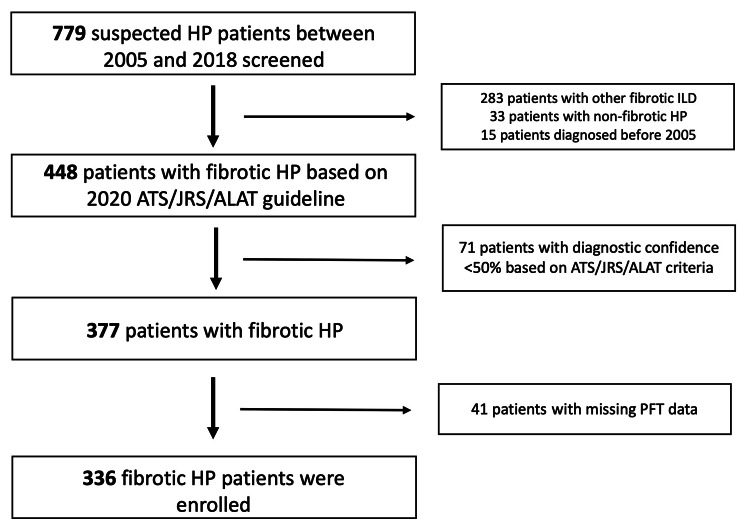
Patient selection

Of 779 patients with suspected f-HP evaluated between January 2005 and December 2020, 448 were compatible with f-HP based on 2020 ATS/JRS/ALAT guideline. Seventy-one and forty-one patients were excluded respectively for diagnostic confidence < 50% and missing baseline PFTs. A total of 336 f-HP patients were included in the final analysis (Fig. 1) with a mean age of 65.3 ± 10.9 years. Approximately half were male and had a history of smoking. Definite diagnosis of f-HP was confirmed in 133 (49.6%) with causative antigen exposures identified in 60% of the total cohort.

**Fig. 2:**
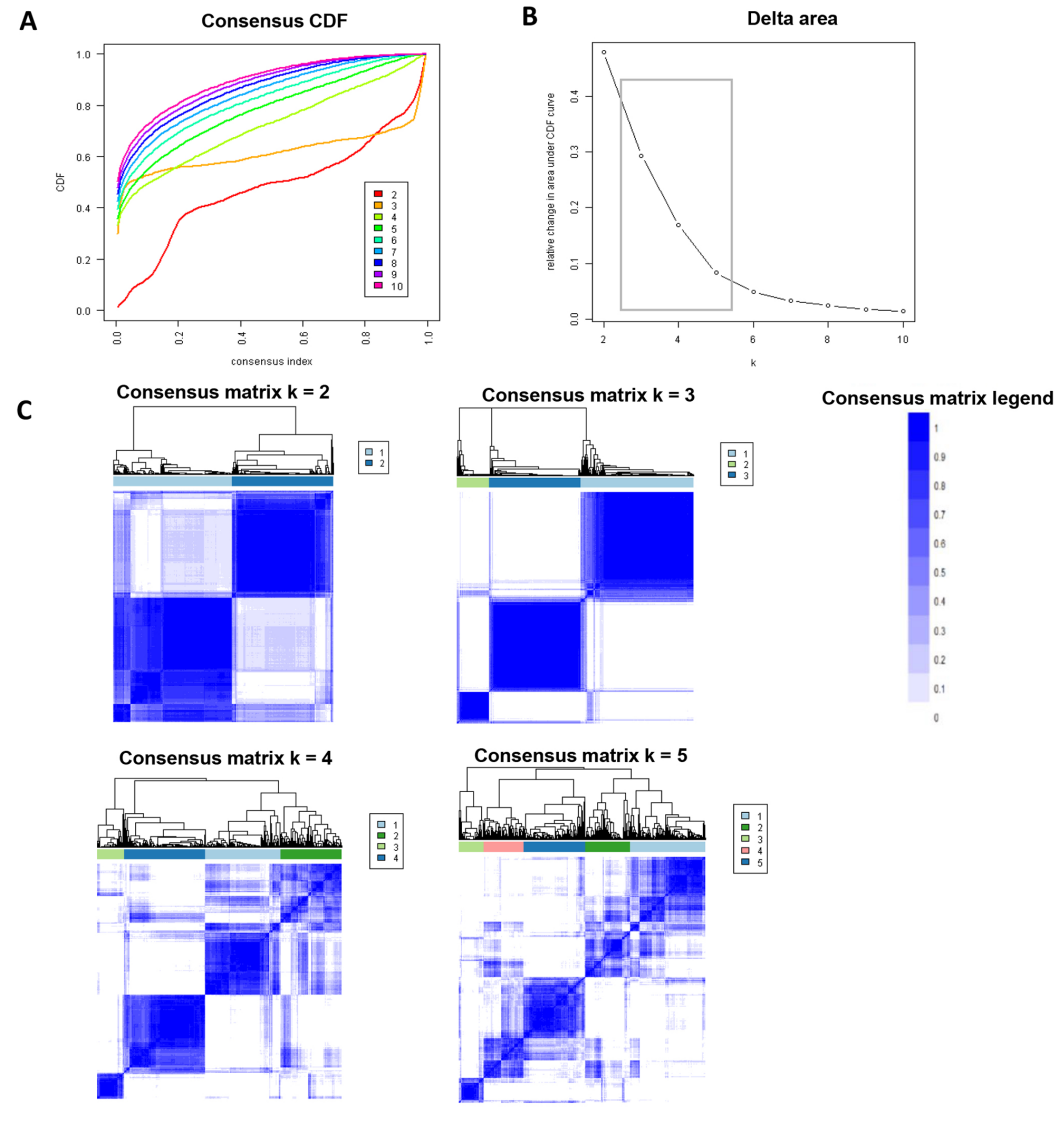
**(A)** CDF plot displaying consensus distributions for each K. Each color represents a specific number of clusters. **(B)** Delta area plot (x-axis (k) signifies the number of clusters). The plot demonstrates relative changes in area beneath the CDF curve with increasing numbers of clusters. **(C)** Consensus matrix heat map depicting consensus values on a white to blue color scale of each cluster

**Fig. 3:**
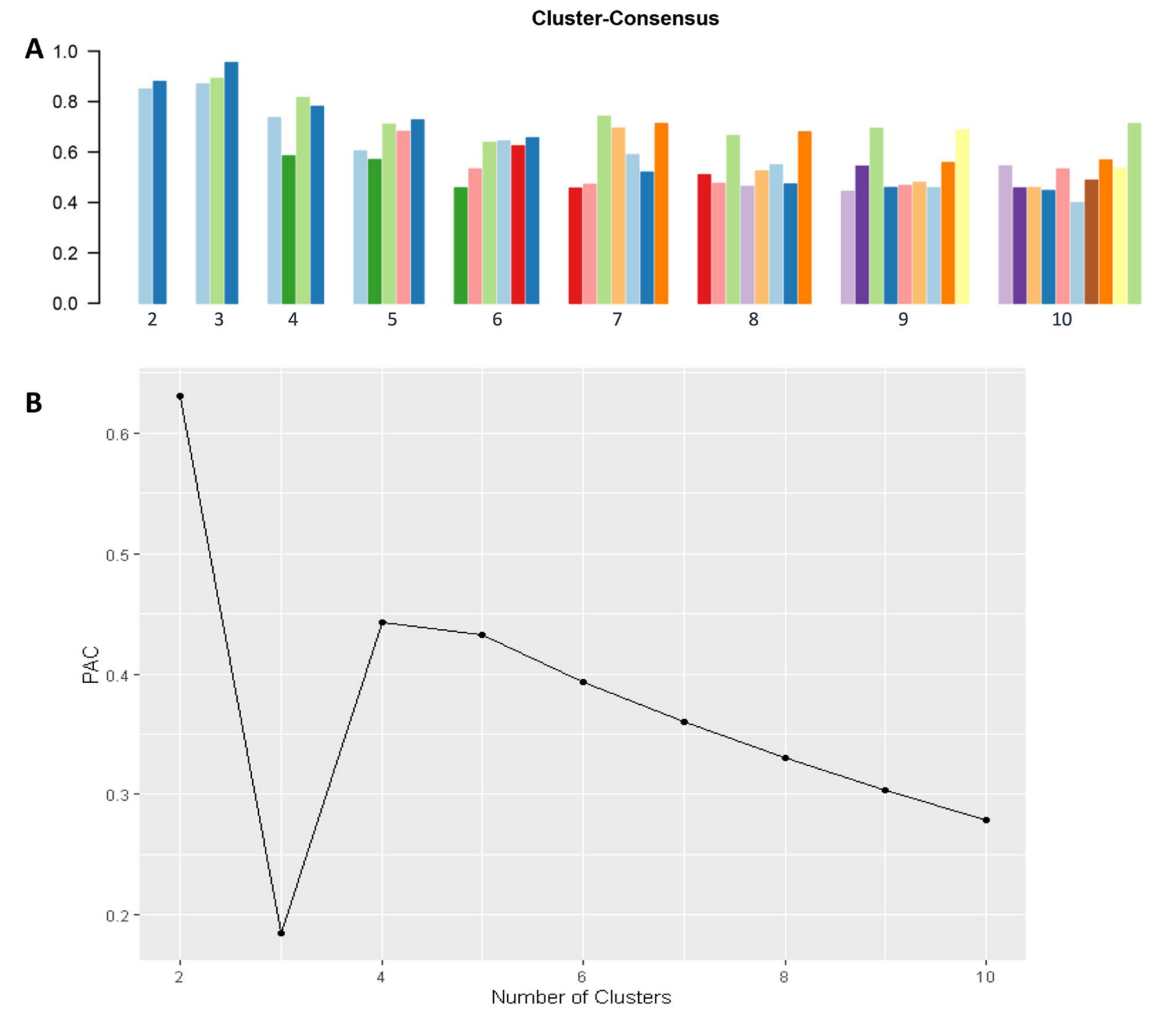
**(A)** The bar plot displays the mean consensus score for different numbers of clusters, where k ranges from two to ten. Each colored bar within a specific number represents an individual cluster from separate clustering simulations. This iterative approach was adopted to evaluate stability and consistency of the clustering results. **(B)** The PAC values assess ambiguously clustered pairs

Consensus clustering analysis was applied to the final set of f-HP patients meeting inclusion criteria. A CDF plot provides the consensus distributions for each cluster (Fig. 2A). A delta area plot shows relative change in the area under the CDF curve (Fig. 2B). The greatest changes in area were identified between k = 3 and k = 5. As shown on the CM heatmap (Fig. 2C, supplementary Figs. 1–9), the ML algorithm identified cluster 3 with distinct borders, demonstrating high cluster stability across repeated iterations. The mean cluster consensus score was highest for three clusters (mean consensus score of 0.90) (Fig. 3A) with favorable low PACs demonstrated for cluster 3 (Fig. 3B). Overall, consensus clustering analysis identified three clinically distinct phenotypes.

**Fig. 4:**
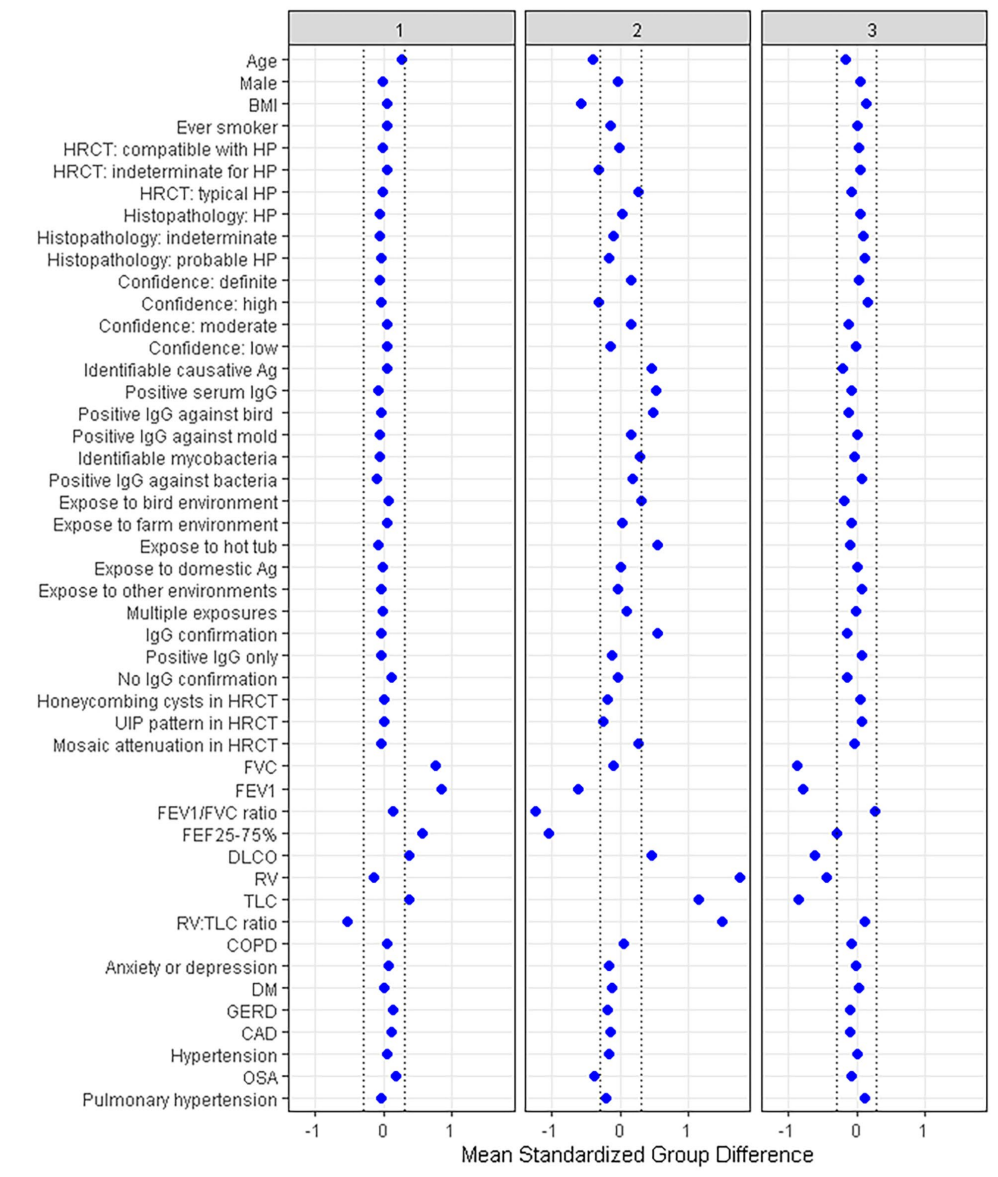
The standardized mean difference plot identifying clinical characteristics of each cluster

Of the 336 f-HP patients, 158 (47.0%), 46 (13.7%), and 132 (39.3%) were classified into clusters 1, 2, and 3, respectively. Baseline characteristics of the three clusters are presented in Table 1. Variables differing among the three included age, BMI, diagnostic confidence, causative antigen identification, baseline PFT findings, and OSA as a comorbidity. The standardized mean difference plot was used to identify key clinical characteristics of each cluster, as presented in Fig. 4.

Cluster 1 were more likely to have preserved pulmonary function defined by only slightly decreased mean FVC (78.2%predicted) and TLC (78.4%predicted), despite being the oldest of the three clusters in terms of age at presentation (mean age 68 ± 9.7 year). Mean DL_CO_ was 55.8% of predicted, comparable to cluster 2 but significantly higher than cluster 3. Cluster 2 had lower mean age (60.9 years) and BMI (27.5 kg/m^2^) with more causative antigen identification (84.8%), particularly to avian and hot tub exposure. PFT findings were also more obstructive with air trapping, lower mean FEV_1_/FVC ratio (0.69), FEV_1_ (57.8%predicted), and FEF_25 − 75%_ (44.5%predicted). Higher mean RV (139.9%predicted), TLC (90.8%predicted), and RV/TLC (151.8%predicted) were also found compared to the other two clusters. Cluster 3 had more severe restriction, with lower mean FVC (51.1%predicted), RV (68.6%predicted), TLC (59.2%predicted), and DL_CO_ (40.5%predicted). Characteristics of the entire cohort and each cluster are presented in Fig. 4; Table 1.

Treatment details are presented in Table 1. With respect to therapeutic interventions, patients in Cluster 2 were more likely not to receive treatment of any kind (26%), including corticosteroid and steroid-sparing agents. Significantly higher antigen avoidance was also observed in this cluster (50%).

**Fig. 5:**
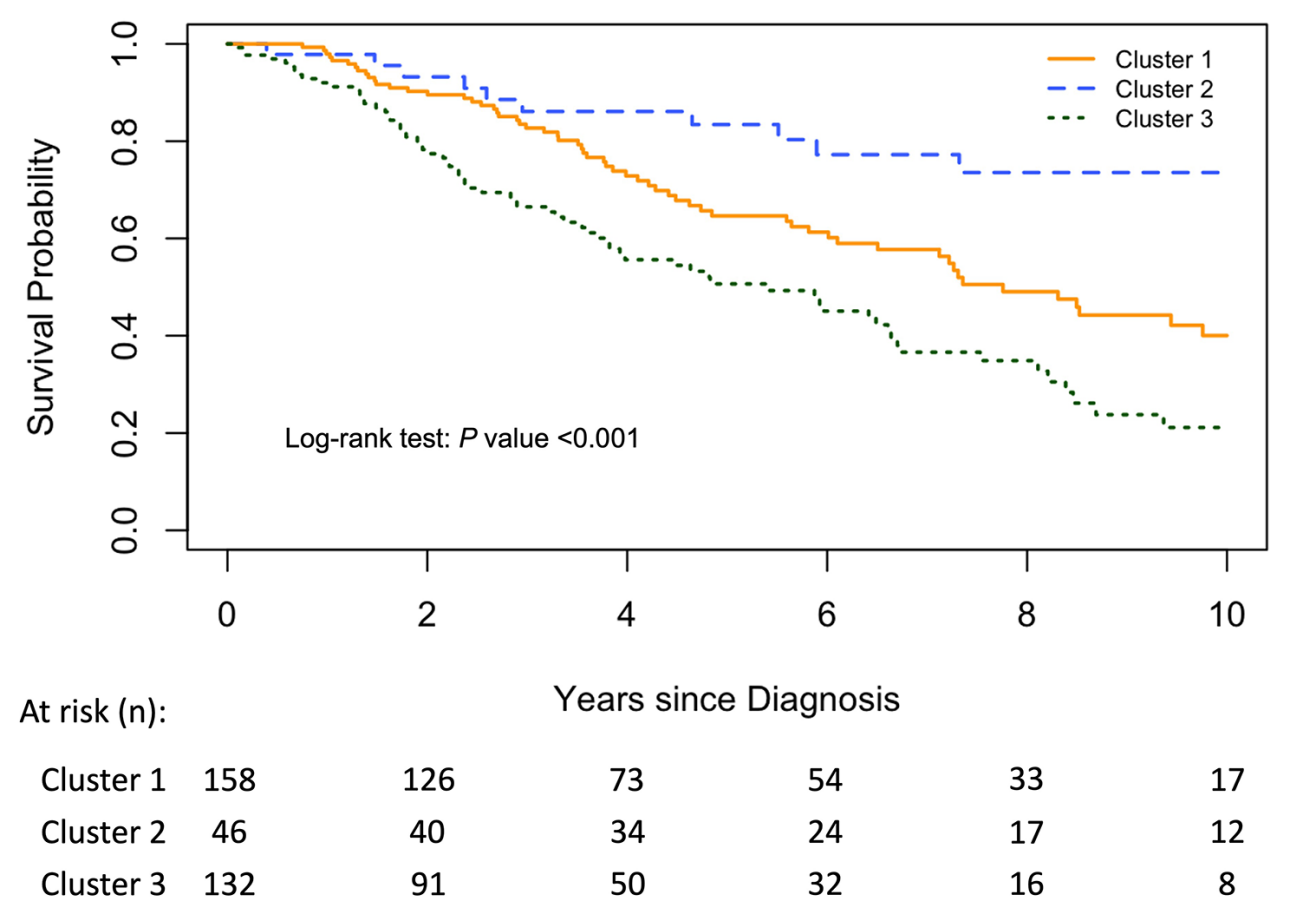
Kaplan-Meier survival curves comparing transplant-free survival among each cluster

Of those in cluster 1, 53 (33.5%) died and 12 (7.6%) underwent lung transplantation. In cluster 2, 10 (21.7%) died and 2 (4.3%) underwent lung transplantation. In cluster 3, 60 (45.5%) died and 11 (8.3%) underwent lung transplantation. When compared to cluster 1, risk of lung transplantation or death was significantly lower for cluster 2 (hazard ratio (HR) 0.42; 95% CI, 0.21–0.82; *P* = 0.01), and significantly higher for cluster 3, (HR 1.76; 95% CI, 1.24–2.48; *P* = 0.001). Kaplan-Meier survival curves for the three clusters are presented in Fig. 5.

## Discussion

Phenotypic characterization resulting in prognostic or differential outcomes has not been previously described in patients with f-HP. Individual clinical parameters have been reported as relevant to predicting outcome (exposure history, lung function, and radiologic findings), though such findings may be heterogenous or present variably among diverse sets of patients [4, 5, 13]. A cluster algorithm approach may identify groups of similar patients using a wide-ranging set of clinical characteristics [6]. A primary advantage of cluster analysis is the potential discovery of new or unexpected disease patterns which may not be intuitive or difficult to characterize due to multifaceted or overlapping presentations. In this study, an unsupervised ML consensus clustering algorithm identified three distinct clusters of f-HP patients based on presenting findings. Key features of each cluster were highlighted by pulmonary function and causative antigen exposure history, despite the inclusion of multiple clinical variables and comorbidities in the analysis. Importantly, the three clusters translated to separate transplant-free survival in the setting of typical treatment or antigen avoidance strategies.

Cluster 1 accounted for most of the f-HP patients included in our cohort (47.0%). Patients in this group had mild restrictive pulmonary physiology with slightly decreased mean FVC and DL_CO_, despite older age at presentation. Mortality or transplant outcomes were observed on average after ten or more years of follow-up. Higher pulmonary function may represent earlier diagnosis, though the subsequently longer survival seen here may represent slower progression or better response to subsequent antigen avoidance or treatment. Similarly, Cluster 3, characterized by more severe restrictive physiology, may also represent more advanced or late-stage disease despite younger age at presentation, as f-HP may present at any age. Baseline FVC and DL_CO_ have been previously described as outcome predictors in f-HP [14, 15]. Notably, UIP HRCT pattern (6 vs. 8%) and honeycombing (18 vs. 21%) were found with similar frequency between the two groups.

Our study found patients in Cluster 2 were uniquely characterized by obstructive physiology on PFTs. The impact of obstruction on outcome or its relation to other clinical characteristics remains unclear in patients with f-HP. Obstruction may be seen in HP as an acute or earlier manifestation of small airways involvement. Mosaic attenuation or expiratory air trapping, typical HRCT findings in f-HP, may also represent small airway involvement with physiologic obstruction [16]. Zuniga et al. found patients with f-HP had improvement in likely small airway-related obstruction, as characterized by a decrease in the phase 3 slope of ultrasonic pneumography, after immunosuppressive treatment [17]. Obstructive physiology might represent active and potentially reversible small airways inflammation or injury responsive to therapy, and perhaps better survival.

Patients in cluster 2 were also younger, had lower BMI, and higher rates of identifiable causative antigen, particularly to avian or hot tub exposure. Younger age, identifiable causative exposure, and antigen avoidance have been previously reported as associated with improved mortality [4, 13, 18]. Our study confirms findings from a previous report demonstrating better survival in patients with history of avian antigen exposure [13]. Since exposure to avian antigens or hot tubs is often easier to identify and avoid, such patients might also have better outcomes. Additionally, compared to clusters 1 and 3, mosaic attenuation occurred more frequently. Honeycombing was also found in 11%, with none having typical or probable UIP HRCT patterns.

As discussed, cluster analysis not only identifies distinctive presenting characteristics inherent to a particular group but may also derive guidance for tailoring appropriate treatment according to associated disease progression or survival outcome. Our study found that patients in Cluster 2 had more favorable outcomes with nearly 30% abstaining from any medical treatment. In contrast, patients in Cluster 3 experienced worse survival despite nearly all receiving initial corticosteroids and half going on to long-term steroid-sparing agents. The earlier use of antifibrotics when meeting criteria for progressive pulmonary fibrosis (as suggested for Cluster 3) may be an appropriate treatment strategy.

Our study has several limitations. First, selection bias is possible with the use of a single tertiary referral center and patients evaluated over a decade or more of clinical experience. Despite the systematic use of recent international consensus criteria to align diagnostic uncertainty, historical practices and their evolution over time may limit the availability of all clinical parameters. Original multidisciplinary team discussions were not documented for all patients; however, extensive clinical, HRCT, and pathological reports were available for defining diagnostic confidence levels according to the 2020 ATS/ERS/JRS/ALAT guideline. Excluded patients who did not have baseline PFTs (*N* = 41) were also younger with a higher proportion of ‘definite’ HP diagnostic confidence levels (supplement Table S1), which might impact the current analyses if included. While a broad range of clinical variables were included in the cluster analysis, there may still be unaccounted or unknown factors that may impact or change current phenotypic characterizations, including timing of symptom onset. Finally, an all-cause mortality endpoint may not entirely represent the direct impact of disease progression from f-HP but contribution from other unrelated comorbidities or complications. We attempted to account for this with the inclusion of selected comorbidities in the clustering model, of which none appeared to be distinguishing.

## Conclusions

We identified three distinct phenotypes of f-HP using an unsupervised machine learning consensus clustering approach. These three clusters, as characterized by pulmonary function testing (mild vs. more severe restriction vs. obstruction) and identifiable antigen exposure history, translated to unique transplant-free survival outcomes.

### Electronic supplementary material

Below is the link to the electronic supplementary material.

Supplementary Material 1

## Data Availability

The datasets used and/or analysed during the current study are available from the corresponding author on reasonable request.
